# Pangolin-derived sarbecoviruses show attenuated pathogenicity and transmissibility compared with SARS-CoV-2 in human ACE2 transgenic hamsters

**DOI:** 10.1128/mbio.01843-26

**Published:** 2026-09-24

**Authors:** Hao-Feng Lin, Ren-Di Jiang, Li-Na Ji, Jin-Wei Zhang, Qian-Chun Gong, Hao-Rui Si, Yuan-Sen Shi, Xin-Hua Lin, Chen Qiu, Jian-Min Li, Mei-Qin Liu

**Affiliations:** 1 The First Affiliated Hospital of Guangzhou Medical University, Guangzhou Laboratory Clinical Base, State Key Laboratory of Respiratory Disease, Guangzhou Medical University 26468, Guangzhou, China; 2 School of Public Health, Nanjing Medical University 572407, Nanjing, China; 3 State Key Laboratory of Genetic Engineering, Greater Bay Area Institute of Precision Medicine (Guangzhou), School of Life Sciences, Zhongshan Hospital, Fudan University 98433, Shanghai, China; 4 School of Life Sciences, Inner Mongolia University, Hohhot, China; 5 State Key Laboratory of Reproductive Medicine and Offspring Health, Jiangsu Animal Experimental Center of Medicine and Pharmacy, Animal Core facility, Key Laboratory of Model Animal, Department of Cell Biology, Collaborative Innovation Center for Cardiovascular Disease Translational Medicine, Nanjing Medical University 12461, Nanjing, China; 6 State Key Laboratory of Respiratory Disease, Guangzhou Medical University 26468, Guangzhou, China; Washington University in St. Louis, St. Louis, Missouri, USA

**Keywords:** pangolin-derived coronaviruses, SARS-CoV-2, pathogenicity, cross-species infection and transmission

## Abstract

Genetic diversity of sarbecoviruses phylogenetically related to SARS-CoV-2 has been identified in Malayan pangolins. However, their potential pathogenicity and risk of cross-species transmission to humans remain largely unknown. In the present study, we performed comparative characterization of SARS-CoV-2 and two pangolin SARS-CoV-2-related viruses, named MpCoV-GD and MpCoV-GX, respectively, in human cell lines and organoids and human ACE2 (hACE2) transgenic hamsters. The cell-to-cell fusion assay showed that the Spike (S) protein of MpCoV-GD induces a stronger membrane fusion than the MpCoV-GX S protein, though weaker than the SARS-CoV-2 S protein. In human cells and organoids, both MpCoV-GX and MpCoV-GD replicated well but less efficiently than SARS-CoV-2. In K18-hACE2 hamsters, MpCoV-GD induced lung pathology comparable to SARS-CoV-2 by showing extensive damage to the alveolar parenchyma, while MpCoV-GX mainly caused alveolar wall thickening due to mild inflammatory cell infiltration. Similar to SARS-CoV-2, the two pangolin coronaviruses were capable of transmitting through direct contact and aerosol exposure, but their infectivity was largely attenuated during aerosol transmission. This study expands our understanding of the biological and infection features of the pangolin-derived sarbecoviruses. It demonstrates that current SARS-CoV-2-related therapeutics and pre-existing immunity can elicit cross-protection against the pangolin sarbecoviruses. Although their pathogenicity and transmissibility are relatively low, our findings suggest the necessity of continuing sarbecovirus surveillance in pangolins and other wildlife for prevention of future disease emergence.

**IMPORTANCE:**

Pangolin-derived sarbecoviruses capable of infecting human cells underscore the role of wildlife as reservoirs for potential zoonotic emergence. MpCoV-GD induces lung pathology comparable to SARS-CoV-2 in hACE2 hamsters, highlighting the need for proactive assessment of novel coronaviruses. Although aerosol transmission of MpCoV-GD and MpCoV-GX is attenuated, their ability to spread via direct contact and airborne routes indicates a tangible risk of cross-species transmission. Importantly, cross-neutralizing activity or inhibition conferred by existing SARS-CoV-2 immunity and therapeutics suggests that current preparedness strategies may help mitigate their impact. These findings reinforce the necessity of sustained surveillance of sarbecoviruses in pangolins and other wildlife to enable early detection and prevention of future outbreaks with significant public health consequences.

## INTRODUCTION

A growing number of emerging zoonoses in the past decades highlight the importance of surveillance of pathogen transmission from wildlife to humans (1). Severe acute respiratory syndrome (SARS) and the coronavirus disease 2019 (COVID-19) are two severe respiratory diseases that posed serious challenges to global health (2, 3). Their causative agents, SARS coronavirus (SARS-CoV) and SARS-CoV-2, are two emerging coronaviruses belonging to the species *Severe acute respiratory syndrome-related coronavirus* (SARSr-CoV), subgenus *Sarbecovirus* (4). Bats are regarded as the probable natural reservoirs of these two viruses, as closely related viruses have been discovered in *Rhinolophus* bats from Asia (5–7). In addition, diverse SARSr-CoVs phylogenetically related to SARS-CoV-2 have been discovered in pangolins smuggled to China, suggesting that these animals may act as important intermediate hosts in the virus transmission (8–10).

The pangolin sarbecoviruses known to date mainly consist of two clusters, which were discovered in smuggled Malayan pangolins (*Manis javanica*) seized by customs of Guangdong and Guangxi Provinces, China, respectively. For each cluster, complete genome sequences of different strains with slight variation have been determined, and the representative strains of both clusters of pangolin sarbecoviruses were successfully isolated in VeroE6 cells, which are referred to as MpCoV-GD and MpCoV-GX in this study. MpCoV-GX and MpCoV-GD share 85.2% nucleotide (nt) identity in genome sequences and 87.7% amino acid (aa) identity in the spike (S) between each other. They share 85.4% and 90.1% nt identity in genome sequence and 92.6% and 90.7% aa identity in spike protein with SARS-CoV-2, respectively (8, 9). Notably, the receptor-binding domain (RBD) of MpCoV-GD was highly identical to that of SARS-CoV-2, differing by seven aa residues (11). Previously, studies showed that MpCoV-GD and MpCoV-GX can efficiently utilize human angiotensin-converting enzyme II (ACE2) as the receptor and replicate in human cells. Both viruses caused lung infection in human ACE2 (hACE2) transgenic mice and can be transmitted through direct contact among infected animals (11–15). It was also reported that pangolins infected with MpCoV-GD exhibited bilateral ground-glass lung opacities which simulate what is observed in COVID-19 patients, suggesting its potential pathogenicity (16). Serological data, although limited, indicate that pangolin infections by sarbecoviruses in Southeast Asia could date back to as early as 2003 (17).

Although previous studies investigated the cross-species transmission risk of the pangolin-origin sarbecoviruses, there is no overall comparison of the two distinct pangolin coronaviruses, MpCoV-GD and MpCoV-GX, in parallel with SARS-CoV-2. In this study, we conducted a comparative analysis of the cellular entry mechanisms, as well as infectivity, transmission, and pathogenicity of these two animal sarbecoviruses, alongside SARS-CoV-2, using multiple human cells, human organoids, and hamsters expressing hACE2. Furthermore, we evaluated the cross-protection provided by SARS-CoV-2-immunized serum and available antiviral agents against both pangolin viruses. This study improves our understanding of pathogenic features of diverse sarbecoviruses circulating in wildlife and provides a more complete picture of their potential risk of cross-species infection in humans.

## MATERIALS AND METHODS

### Cells and viruses

Cercopithecus aethiops kidney cells (VeroE6, ATCC CRL-1586), human lung adenocarcinoma cells (Calu-3, ATCC, CVCL-0609), and human colon cells (Caco-2, ATCC, CVCL-0025) were obtained from the National Virus Resource Centre (Wuhan, China). Huh7 cells, derived from human liver tissue, were kindly provided by Prof. Linfa Wang (Duke-NUS Medical School, Singapore). These cell lines were cultured in Dulbecco’s modified Eagle medium (DMEM; Gibco) or DMEM/nutrient mixture F-12 (DMEM/F-12; Gibco) supplemented with 10% fetal bovine serum (FBS; Gibco) at 37°C with 5% CO_2_. All cell lines were routinely tested and maintained mycoplasma-free. MpCoV-GX (GenBank accession no. MT072864.1) was offered by Prof. Yigang Tong (8), while SARS-CoV-2 (IVCAS 6.7512) was isolated from a COVID-19 patient sampled in 2019 (18). MpCoV-GD was recovered from a full-length cDNA clone constructed by yeast genetic engineering technology in this study. A rabbit polyclonal antibody against the nucleocapsid protein (NP) of bat SARS-related coronavirus (SARSr-CoV) Rp3 which shares 90% amino acid identity with SARS-CoV-2 was developed in-house.

### Construction of MpCoV-GD full-length cDNA clone

The genomic sequence of MpCoV-GD (GenBank accession no. MT121216.1) was fragmented into 15 segments and synthesized at Sangon Biotech, Tsingke Biotech, and Genscript Biotech Corporation. The full-length genomic cDNA was synthesized and assembled according to a previously published protocol (19). In brief, VL6-48 yeast cells were cultured overnight in YEPD medium at 30°C to generate protoplasts. Virus genomic DNA and shuttle vector PGF fragments were mixed with yeast protoplasts and plated on SD-His plates, followed by incubation at 30°C for 72 h. Colonies positive for all junctions were cultured in 5 mL of SD-His broth, and DNA extraction was performed using the Yeast Plasmid Mini Kit (Omega), as per the manufacturer’s instructions. Positive clones were transformed into EPI300 Super Competent Cells and cultured in LB broth. Virus genomic DNA was then extracted using a BAC/PAC DNA Kit, as per the manufacturer’s protocol. For transfection into Vero E6 cells in six-well plates, 5 μg of plasmid DNA was combined with 10 μL of P3000 reagent and 15 μL of Lipofectamine 3000 reagent (Invitrogen). Cytopathic effects were observed 5 days post-transfection (dpt), after which the supernatant was transferred to VeroE6 cells cultured in T75 flasks for further amplification and titered by 50% tissue culture infective dose (TCID_50_) assay.

### Cell-cell fusion assay

293T cells were transfected with plasmids encoding the S proteins of SARS-CoV-2, MpCoV-GX, or MpCoV-GD, along with the GFP_1-7_ RLN plasmid. VeroE6 cells were transfected with the GFP_8-11_ RLC plasmid. This split GFP plasmid was generously provided by Prof. Youchun Wang. Both types of cells were incubated at 37°C with 5% CO_2_ for 24 h. The transfected 293T and VeroE6 cells were then mixed at a 1:1 ratio and seeded into 48-well plates. GFP and *Renilla* luciferase fluorescence were measured 8 h after mixing. GFP signals were captured using the AMF4300 Evos FL Cell Imaging System (Life Technologies, Carlsbad, CA, USA). The EnduRen live-cell substrate (Promega, E6481, WI) and Ensight system (PerkinElmer) were employed for luciferase activity detection.

### Attachment and internalization assays

For the virion attachment and internalization assay, we followed the procedures described in our previous study (20). Briefly, the indicated cells were seeded in 48-well plates and then infected with SARS-CoV-2 and MpCoV-GD/-GX at a multiplicity of infection (MOI) of 0.1. The infected cells were incubated at 4℃ for 1 h to permit virus attachment while preventing virus entry. Subsequently, the supernatant was removed, and the cells were washed three times with the pre-chilled PBS. The cell-bound virus was isolated using TRIzol reagent. The viral RNA was evaluated by RT-qPCR. To analyze viral internalization, the infected cells were placed at 4℃ for 1 h, washed three times with PBS, and incubated in pre-warmed DMEM at 37°C. After 1 h, cells were treated with 1 mg/mL pronase dissolved in PBS at 37°C for 10 min to remove non-internalized virus particles from the cell surface. Upon three washes with PBS, the cells were harvested for viral detection. To be in keeping with the infective dose unit, the titer of SARS-CoV-2, MpCoV-GD, and MpCoV-GX was determined by TCID_50_ assay, and the RNA from gradient dilution virus stocks with known titer was used as a standard control to correlate Ct value and virus titers.

### Immunofluorescence staining

The growth curve of SARS-CoV-2 and MpCoV-GD/GX was assessed using VeroE6, Huh7, Caco-2, and Calu-3 cell lines. Cells were seeded in 48-well plates at 90% confluence and inoculated with the viruses at a MOI of 0.01. After inoculation, cells were washed three times with PBS and maintained in DMEM supplemented with 2% FBS. At 24 hours post-infection (hpi), the cells were fixed with 4% paraformaldehyde at 4°C for 24 h and then permeabilized with 0.1% Triton X-100. Blocking was performed using PBS containing 5% bovine serum albumin, followed by incubation with anti-Rp3 nucleocapsid protein (Np) polyclonal antibody. After washing with PBS, cells were incubated with Cy3-conjugated anti-rabbit IgG and stained with DAPI. Fluorescence images were captured and analyzed using the AMF4300 Evos FL Cell Imaging System (Life Technologies, Carlsbad, CA, USA).

### Protease inhibition

293T cells stably expressing human ACE2 (293T-hACE2) were transfected with plasmids expressing Furin, transmembrane protease serine 2 (TMPRSS2), and Cathepsin L. Two hours prior to infection, the corresponding protease inhibitors (50 μM), decanoyl-Arg-Val-Lys-Arg-chloromethyl ketone (decRVKRcmk), camostat, and E64D, or blank solvents DMSO were added. The cells were then infected with SARS-CoV-2, MpCoV-GD, and MpCoV-GX. At 24 hpi, the supernatant was used for viral detection, while the cells were fixed with 4% paraformaldehyde for virus N protein analysis.

### Human organoid infection and immunofluorescent staining

The culture system for human organoids was previously established and modified (21). For each type of tissue, the organoids were derived from a single donor. For virus infection, organoids were extracted from Matrigel using a cell recovery solution (Corning, 354253) at 4°C for 1 h. To enhance viral entry and infection, the organoids were gently dissociated to expose their lumen through either digestive or mechanical disruption with 1× TrypLE Select (Gibco, A1217701). The organoids were then infected with viruses at MOI ranging from 0.1 to 1 and incubated at 37°C for 3 h in a CO_2_ incubator maintained at 5% concentration. Following infection, the organoids were washed twice with PBS before being resuspended in Matrigel. The resuspended organoids were then placed in a CO_2_ incubator at 37°C for 30 min to allow Matrigel solidification. Finally, the virus-infected organoids were collected at various time points during culture.

For immunofluorescence staining, Matrigel was dissolved using a cell recovery solution. After separation from the Matrigel, the organoids were fixed at 4°C with 4% PFA for 1 h, permeabilized with 0.25% Triton X-100 (Sigma, T9284) in PBS for 15 min and subsequently blocked with 5% BSA (BBI, A600332) for 1 h. The organoids were then incubated overnight at 4°C with the primary antibody, followed by three times of washing with PBST (PBS containing 0.1% Tween-20). Subsequently, the organoids were incubated with the corresponding secondary antibody and DAPI (Roche, 10236276001) at room temperature for 1 h, followed by PBST washing. Confocal microscopy (Leica, STELLARIS 8) was used to visualize the organoids. Anti-Rp3-NP (1:1,000) and anti-E-cadherin (1:100, BD Biosciences, 610180) were used as the primary antibodies. FITC-conjugated Affinipure Goat Anti-Mouse IgG (H + L) (1:200, Proteintech, SA00003-1) and Goat Anti-Rabbit IgG H&L (Cy3) (1:200, Abcam, ab6939) were used as the secondary antibodies.

### RNA extraction and quantitative real-time PCR

The cell culture suspension was heat-inactivated, and viral RNA in the samples was quantified using a one-step qRT-PCR assay. Viral RNA was extracted with the Virus DNA/RNA Extraction Kit (Vazyme, RM501) and used as the template for qRT-PCR amplification with the HiSxript II One-Step qRT-PCR SYBR Green Kit (Vazyme, Q221). The following primer pairs were used for targeting the SARS-CoV-2 nucleocapsid gene and the MpCoV-GD/-GX N gene: SARS-CoV-2 Np-qF1 (5′-GAGCTACCAGACGAATTCGTGGT-3′)/Np-qR1 (5′-CTCAAGTGTC TGTGGATCACG-3′); MpCoV-GD Np-qF1 (5′- GTGATGCTGCTCTTGCTTTGC-3′)/Np-qR1 (5′- AGCGGATTTCTTAGTGACAGTTTG-3′); MpCoV-GX Np-qF1 (5′- TACCAGACGAGTTCGTGGTGGT-3′)/Np-qR1 (5′- ATTGCGGGTGCCAATGTGATC-3′). PCR was performed according to the manufacturer’s instructions.

### Animal infection experiments

The H11-K18-hACE2 transgenic golden hamster strains were generated by injecting a two-cell embryo with CRISPR–Cas9 using H11 sgRNA and the specific process as previously described (22). Animals were inoculated with the virus under appropriate anesthesia, and efforts were made to minimize any potential pain and distress. Eight- to twelve-week-old male and female K18-hACE2 hamsters were randomly divided into different groups. (i) Survival monitoring group: hamsters were mock infected (*n* = 8) or intranasally infected with 1 × 10^5^ TCID_50_ SARS-CoV-2 (*n* = 10), MpCoV-GD (*n* = 8), or MpCoV-GX (*n* = 8). Body weight, clinical symptoms, and viral shedding were monitored daily up to 7 days post-infection (dpi). (ii) Hamsters (*n* = 5) in each group were infected with the above three viruses, and tissues were collected at 2 and 4 dpi for virus titration and pathological analysis. (iii) For the direct contact transmission experiment, hamsters were intranasally infected with 1 × 10^5^ TCID_50_ of SARS-CoV-2 (*n* = 9), MpCoV-GD (*n* = 8), or MpCoV-GX (*n* = 8). Twenty-four hours later, each inoculated animal (index) was co-housed with one naive hamster (contact). Body weight and viral shedding were monitored daily up to 4 dpi. Tissues of all hamsters were collected at 4 dpi for virus titration and pathological analysis. (iv) For the aerosol transmission study, donor hamsters were anesthetized with isoflurane and intranasally inoculated with 1 × 10^5^ TCID_50_ of SARS-CoV-2 (*n* = 4), MpCoV-GD (*n* = 3), or MpCoV-GX (*n* = 4). Aerosol contact hamsters with identical numbers as those in the infection group were placed into the respective aerosol transmission cages containing infected hamsters. Viral shedding was monitored daily up to 4 dpi. Tissues of all hamsters were collected at 4 dpi for virus titration and pathological analysis.

### Determination of virus titers via plaque assay

VeroE6 cells were seeded in 24-well plates 1 day prior to use. Hamster tissues were homogenized in DMEM, and the virus was serially diluted 10-fold. The cells were inoculated with the tissue dilutions for 1 h. After inoculation, the supernatant was removed, and the cells were incubated with 0.9% methylcellulose for additional 5 days. Plaque-forming units (PFU) were counted to calculate the viral titers.

### Histopathology and IFA

Tissue specimens were fixed in 4% paraformaldehyde, routinely processed, paraffin-embedded, and sectioned serially at 3.5 μm thickness. For conventional histopathological assessment, sections were stained with hematoxylin and eosin (H&E) to evaluate general tissue morphology. For IFA, paraffin-embedded sections were deparaffinized, rehydrated through a graded ethanol series, and subjected to heat-induced antigen retrieval in EDTA buffer. After extensive washing with PBS containing 0.02% Triton X-100, sections were blocked with 5% bovine serum albumin (BSA) at room temperature to minimize nonspecific antibody binding. Sections were then incubated overnight at 4°C with primary antibodies, comprising an in-house-generated rabbit polyclonal antibody Rp3-Np (1:1,000) and a commercial mouse monoclonal antibody against NeuN (1:200; Servicebio). Following thorough washing with PBS, secondary primary antibodies were detected with species-specific secondary antibodies: Cy3-conjugated goat anti-rabbit IgG (1:200; Abcam) for viral antigen visualization and Alexa Fluor 488-conjugated goat anti-mouse IgG for neuronal labeling. Cell nuclei were counterstained with 4′,6-diamidino-2-phenylindole (DAPI; Beyotime) at a 1:100 dilution. Fluorescence images were captured using a Pannoramic MIDI digital slide scanner (3DHISTECH) for panoramic overviews and an FV1200 laser scanning confocal microscope (Olympus) for high-resolution imaging.

### Plaque reduction neutralization test

The monoclonal antibody REGN10933 (Casirivimab, HY-P99341) and REGN10987 (Imdevimab, HYP99342) were procured from MedChemExpress. S309 (Anti-SARS-CoV-2 S antibody, ATMA10183Mo) was procured from Atagenix. The sera of mice immunized with SARS-CoV-2 inactivated vaccine (IAV) and the convalescent sera of patients were obtained as previously described (23, 24). The PRNT_50_ values were determined to assess the neutralizing titers of SARS-CoV-2 monoclonal antibody, IAV immune mouse sera, and COVID-19 patient convalescent sera against SARS-CoV-2 and MpCoV-GD/-GX. Briefly, VeroE6 cells were seeded into 24-well plates 1 day prior to use. Serum samples or antibodies were serially diluted in six gradients and incubated with 100 PFU of SARS-CoV-2 or MpCoV-GD/-GX at 37°C for 0.5 h. The mixtures were then added to VeroE6 cells and incubated at 37°C for an additional 1 h. The inoculum was removed, and the cells were incubated with 0.9% methylcellulose for 5 days. Plaque counts were used to calculate the PRNT_50_.

### Antiviral assays

Remdesivir (Selleck, S8932) and Molnupiravir (Selleck, S8969) were purchased from Selleck Chemicals. Antiviral efficacy assays were performed in VeroE6 cells. Cells were plated in 48-well plates and cultured overnight, and 2 h before infection, the culture supernatant of VeroE6 cells was discarded and replaced with a DMEM medium containing different concentration gradient drugs. Then, cells were infected with the indicated virus at an MOI = 0.01 for 1 h. After discarding the supernatant, the cells were washed twice with DMEM, and the medium was changed to a DMEM medium (2% FBS) containing the corresponding concentration of the drug. At 24 hpi, the cell supernatant was collected for viral RNA detection.

## RESULTS

### Characterization of cell entry mechanism of pangolin-origin sarbecoviruses

**Fig 1:**
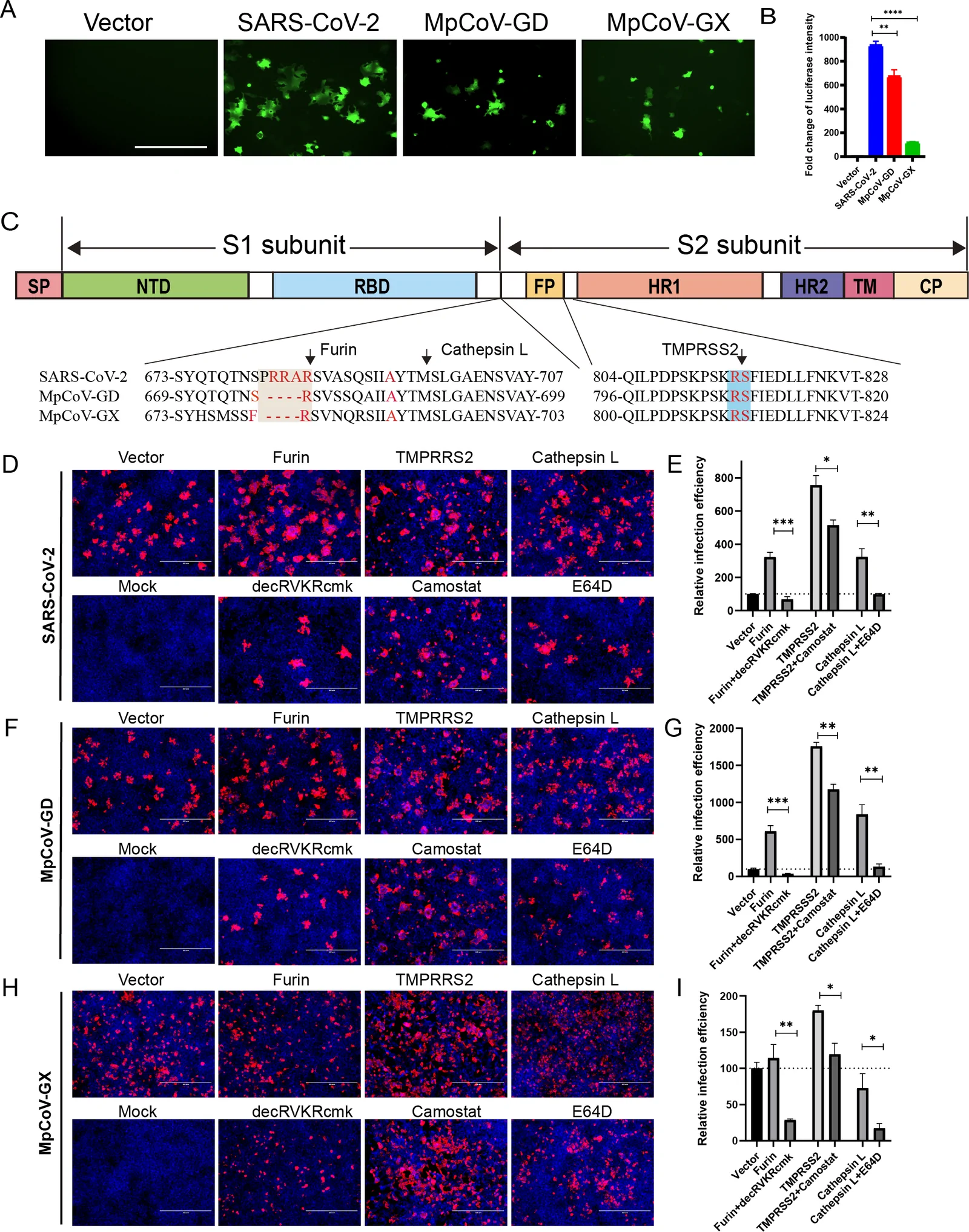
Comparison of the cell entry efficiency of SARS-CoV-2 and the two pangolin SARS-CoV-2-related coronaviruses. (**A**) 293T cells were co-transfected with plasmids expressing S protein and GFP_1–7_RLN, whereas Vero E6 cells were transfected with the GFP_8–11_RLC reporter plasmid. At 24 h post-transfection (hpt), transfected 293T and Vero E6 cells were combined at a 1:1 ratio. GFP fluorescent signals were captured 8 h following cell co-culture. (**B**) Lysates from the co-cultured cells described in panel **A** were collected to quantify luciferase activity as a readout of cell fusion efficiency. (**C**) Sequence alignment of the spike protease cleavage site among SARS-CoV-2, MpCoV-GD, and MpCoV-GX. (**D**) 293T-hACE2 cells were transfected with plasmids encoding furin, TMPRSS2, or cathepsin L. At 24 hpt, cells were pre-treated with 50 μM of the corresponding protease inhibitors (decRVKRcmk, Camostat, and E64D) for 2 h before infection with SARS-CoV-2, MpCoV-GD (**F**), and MpCoV-GX (**H**) at an MOI of 0.1. At 16 hpi, cells were fixed and subjected to immunofluorescence staining targeting viral N protein. Mock control cells were treated with an equal volume of DMSO vehicle alone (red, N protein; blue, cell nuclei). (**E**) Cell culture supernatants harvested from the SARS-CoV-2, MpCoV-GD (**G**), and MpCoV-GX (**I**) infected cells as shown in panels **D**, **F**, and **H** were used to quantify viral copy numbers. The relative infection efficiency was determined as the ratio (% value) of the viral copy numbers in the supernatant of cells transiently expressing Furin, TMPRSS2, or Cathepsin L to those in cells transfected with the vector. All quantitative data are displayed as the mean ± standard error of the mean (SEM). Statistical comparisons in panel **C** were performed using one-way analysis of variance (ANOVA). Asterisks indicate statistically significant differences: *P < 0.05, **P < 0.01, ***P < 0.001, ****P < 0.0001. Groups lacking asterisks exhibited no statistically significant differences.

Previous studies have demonstrated that the fusion capacity of the coronavirus S protein is a key indicator of viral infectivity (25). To evaluate the membrane fusion capacity of the S proteins from pangolin sarbecoviruses, we transfected 293T cells with the plasmids encoding their S proteins and GFP_1-7_RLN, while GFP_8–11_RLC was introduced into VeroE6 cells. Twenty-four hours after transfection, a co-culture system with an equal ratio of these two cell types was established. Following an incubation for 8 h, we assessed the S protein’s ability to induce cell membrane fusion by analyzing GFP and luciferase expression levels. The results indicated that the SARS-CoV-2 S protein exhibited the strongest ability to induce cell membrane fusion, followed by MpCoV-GD and then MpCoV-GX (Fig. 1A and B).

Both MpCoV-GD and MpCoV-GX are able to use ACE2 as the receptor for cell entry (26). In addition to receptor proteins on the cell membrane, the surface proteases of host cells, which play a crucial role in membrane fusion or endocytosis, also contribute to viral entry. For effective membrane fusion, the coronavirus S protein requires priming through proteolytic cleavage at two sites. The first cleavage site is at the S1/S2 boundary, and the second is located at the S2′ site in the S2 subunit (27, 28). For SARS-CoV, both cleavage sites are processed by proteases in the target cell (29, 30). For SARS-CoV-2, the S1/S2 boundary is cleaved by furin in the virus-producing cells and target cells, while cleavage at the S2′ site still requires target-cell proteases. TMPRSS2 and cathepsin L are two key host proteases involved in the activation of the virus spike protein. TMPRSS2-mediated activation occurs at the plasma membrane, while cathepsin-mediated activation takes place within endolysosomes (31). Alignment of the amino acid sequence revealed a lack of a polybasic furin cleavage site at the S1/S2 boundary of the two pangolin sarbecoviruses (MpCoV-GD and MpCoV-GX), but they possessed the TMPRSS2 and cathepsin L cleavage sites in the S2 (Fig. 1C).

To investigate the effects of host proteases on virus entry, we overexpressed furin, TMPRSS2, and cathepsin L in 293T-hACE2 cells. Two hours before infection, the cells were treated with the corresponding protease inhibitors decanoyl-Arg-Val-Lys-Arg chloromethyl ketone (decRVKRcmk), camostat mesylate, and Aloxistatin (E64D), or with blank solvents. Subsequently, the cells were infected with SARS-CoV-2, MpCoV-GD, or MpCoV-GX 24 h post transfection. The overexpression of the three proteases significantly enhanced the infectivity of SARS-CoV-2 and MpCoV-GD in 293T-hACE2 cells, while the effect on MpCoV-GX was much less pronounced. By contrast, treatment with the inhibitors attenuated infection by all three viruses in 293T-hACE2 cells (Fig. 1D through F), indicating that all the three viruses need host proteases for the infection and the endocytosis route plays a major role.

### Comparative characterization of MpCoV-GD, MpCoV-GX, and SARS-CoV-2 infection in human cell lines

**Fig 2:**
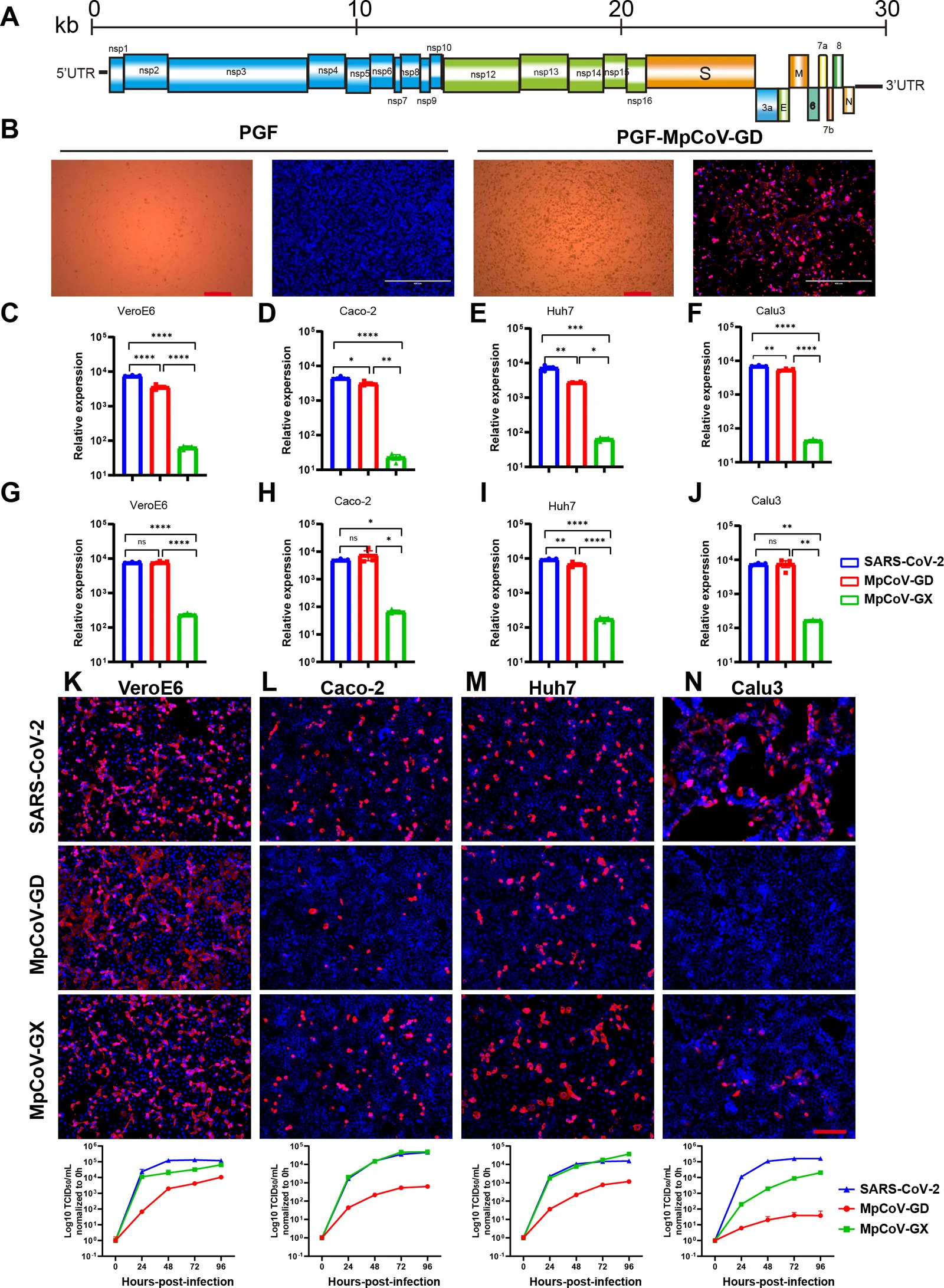
Comparison of attachment, internalization, and replication of SARS-CoV-2, MpCoV-GD, and MpCoV-GX in human cells. (**A**) Schematic of genome structure of MpCoV-GD. (**B**) Full-length cDNA clone of MpCoV-GD was constructed by yeast genome engineering. CPE and virus NP signals were observed in VeroE6 cells transfected with full-length cDNA at 5 days after transfection. The attachment capacity of SARS-CoV-2, MpCoV-GD, and MpCoV-GX on VeroE6 cells (**C**), Caco-2 cells (**D**), Huh7 cells (**E**), and Calu-3 cells (**F**) was assessed. SARS-CoV-2, MpCoV-GD, and MpCoV-GX were incubated with the specified cells at 4°C for 1 h, followed by cell collection for viral detection. The internalization capacity of SARS-CoV-2, MpCoV-GD, and MpCoV-GX on VeroE6 cells (**G**), Caco-2 cells (**H**), Huh7 cells (**I**), and Calu-3 cells (**J**) was also evaluated. After initial incubation at 4°C for 1 h, the cells were further incubated at 37°C for an additional hour. Virions attached to the cell surface without internalization were removed using proteinase, after which the cells were harvested for viral load assessment. VeroE6 (**K**), Caco-2 (**L**), Huh-7 cells (**M**), and Calu-3 cells (**N**) were infected with SARS-CoV-2, MpCoV-GD, and MpCoV-GX (MOI = 0.01). Immunofluorescence assay was performed at 24 hpi. Viral antigens, which were shown in red fluorescence, were stained with a rabbit polyclonal antibody against SARS-CoV-2 NP as the primary antibody, followed by Cy3-conjugated Affinipure Goat Anti-Rabbit IgG(H + L). Cell nuclei were stained with DAPI and shown in blue. Scale bar is 200 μm. Multi-point supernatants were taken at 0, 24, 48, 72, and 96 hpi. Viral loads of SARS-CoV-2, MpCoV-GD, and MpCoV-GX in the four cell lines were determined by RT-qPCR and are shown correspondingly. To be in keeping with the infectious dose unit, the titer of SARS-CoV-2, MpCoV-GD, and MpCoV-GX was determined as TCID_50_ assay, and the RNA from gradient dilution virus stocks with known titer was used as a standard control to correlate *C*_T_ value and viral titer of viruses. The errors are presented as mean ± SEM. Statistical significance was measured by one-way ANOVA (**C**). Asterisks denote significant differences (*P < 0.05, **P < 0.01, ***P < 0.001, ****P < 0.0001); groups without asterisks show no statistical significance. The red scale bar is 200 μm, and the black scale bar is 400 μm.

The genomes of MpCoV-GD and MpCoV-GX exhibit 90.1% and 85.4% overall nucleotide identity with SARS-CoV-2, respectively, while their S protein shows 90.7% and 92.6% amino acid identity to SARS-CoV-2, respectively (8, 9). Previous studies using pseudoviruses have demonstrated that MpCoV-GD and MpCoV-GX have a cellular infectivity spectrum similar to that of SARS-CoV-2 (32). To validate the pseudovirus-based results as well as to characterize their infection properties at different steps during the virus life cycle, we constructed an infectious cDNA clone of MpCoV-GD from VeroE6 cells transfected with a full-length cDNA clone. CPE was observed after 5 dpt (Fig. 2A and B). Then, we performed attachment, internalization, and one-step growth curve assay in VeroE6, Caco-2, Huh7, and Calu-3 cells infected with SARS-CoV-2, MpCoV-GD, and MpCoV-GX. Virus attachment experiments revealed that SARS-CoV-2 had a significantly higher attachment capacity in VeroE6 (Fig. 2C), Caco-2 (Fig. 2D), Huh7 (Fig. 2E), and Calu-3 (Fig. 2F) than MpCoV-GD and MpCoV-GX. Among the two pangolin coronaviruses, MpCoV-GD showed a higher attachment capacity than MpCoV-GX. Except for Huh7 cells, there were no statistically significant differences in the internalization of SARS-CoV-2 and MpCoV-GD in VeroE6, Caco-2, and Calu-3 cell lines, while the internalization level of MpCoV-GX was significantly lower than SARS-CoV-2 and MpCoV-GD in all four cell lines (Fig. 2G through J). One-step growth curve analysis demonstrated comparable replication levels of SARS-CoV-2 and MpCoV-GX in VeroE6, Caco-2, and Huh7 cells, but not in Calu-3 cells. MpCoV-GD, however, displayed lower replication levels than SARS-CoV-2 and MpCoV-GX across all tested cell lines (Fig. 2K through N). These findings suggested superior capabilities of SARS-CoV-2 in attachment, internalization, and replication compared to the pangolin-derived sarbecoviruses. Despite the higher attachment and internalization capacity of MpCoV-GD, its overall replication level remained lower than that of MpCoV-GX.

### Infectivity of SARS-CoV-2, MpCoV-GD, and MpCoV-GX in human organoids

**Fig 3:**
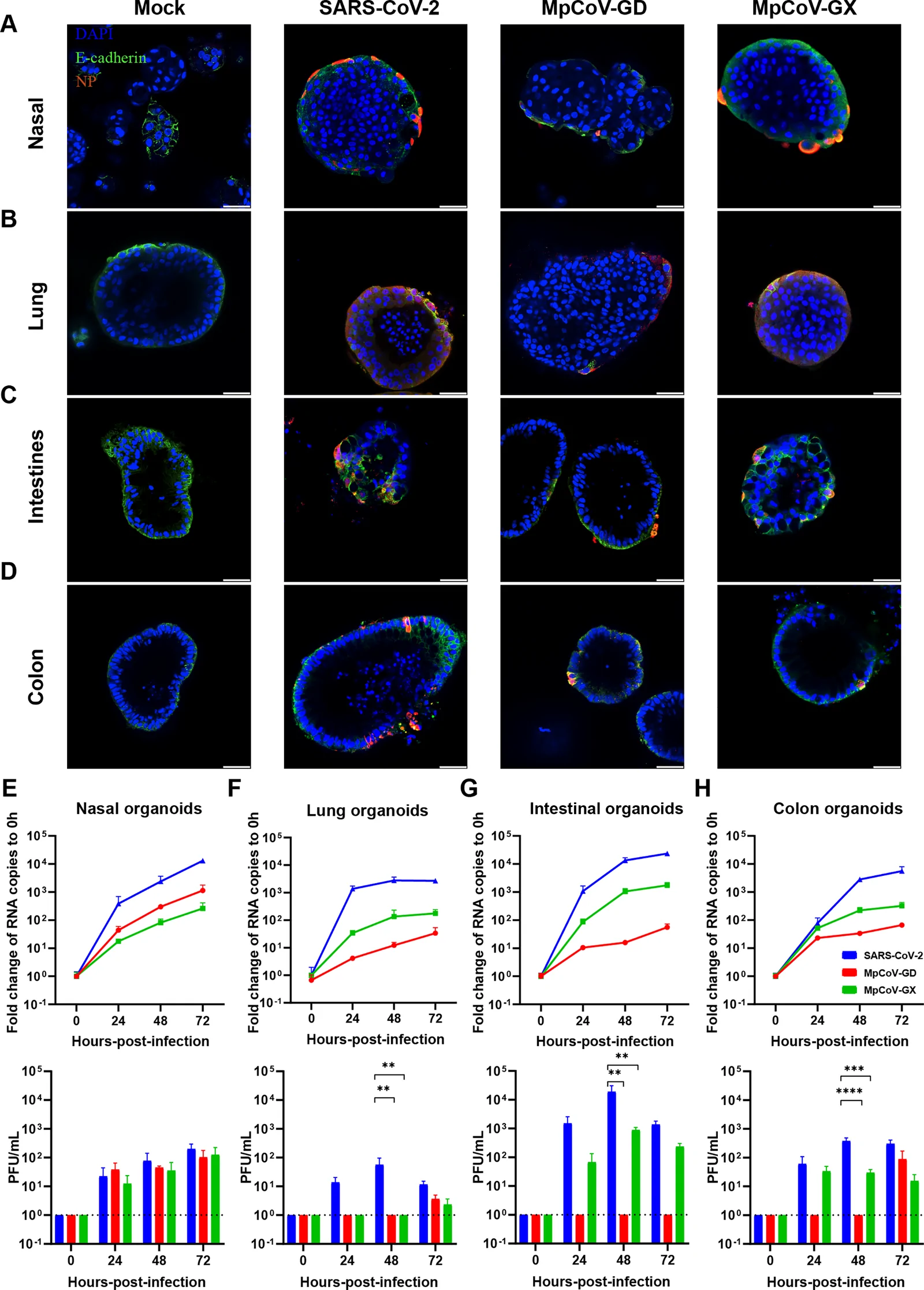
Infection and replication of SARS-CoV-2, MpCoV-GD, and MpCoV-GX in human organoids. Co-staining of SARS-CoV-2, MpCoV-GD, and MpCoV-GX NP (red) and E-Cadherin (green) in human nasal organoids (**A**), lung organoids (**B**), intestinal organoids (**C**), and colon organoids (**D**) at 48 hpi. (scale bar, 40 μm). Replication of SARS-CoV-2, MpCoV-GD, and MpCoV-GX in human nasal organoids (**E**), lung organoids (**F**), intestinal organoids (**G**), and colon organoids (**H**), as analyzed by qRT-PCR and plaque formation assay, respectively, in cell supernatants (top panel) and viral titers (bottom panel). The dotted line indicates the limit of detection in the plaque assay. The errors are presented as mean ± SEM. Statistical significance was measured by two-way ANOVA. Asterisks denote significant differences (**P < 0.01, ***P < 0.001, ****P < 0.0001); groups without asterisks show no statistical significance.

The respiratory and enteric tracts are the primary target organs for coronavirus replication and transmission (33). In addition, coronaviruses have been detected in fecal samples collected from pangolins, suggesting that the gastrointestinal tract may also represent a potential site of viral infection or shedding. To further investigate the potential virus transmission route, we compared the infectivity of SARS-CoV-2, MpCoV-GD, and MpCoV-GX in human respiratory and intestinal organoids. All the viruses can infect and replicate in the four types of human organoids (Fig. 3A through D) with SARS-CoV-2 showing higher replication efficiency than the two pangolin viruses (Fig. 3E through H, top panel) in genomic copy numbers. MpCoV-GX showed better replication than MpCoV-GD except in nasal organoid. All three viruses released viral particles in supernatant in a similar titer in nasal organoids but differently in the other three types of organoids. SARS-CoV-2 particles were detected in all the other three organoids from 24 hpi, with the highest titers in intestinal organoid and the lowest in lung organoid (Fig. 3E through H, lower panel). MpCoV-GX live viruses were detected in intestinal and colon organoids from 24 to 72 hpi but not detected in lung organoid until 72 hpi. MpCoV-GD viral particles were detected in lung and colon organoids at 72 hpi, not detectable in intestinal organoid. These results indicate that the two pangolin viruses have an attenuated infection and replication capacity than SARS-CoV-2 in human organoids. In consistent with the above results of infection in cell lines, MpCoV-GD does not adapt to human cells during viral replication although it has a superior attachment capacity than MpCoV-GX.

### Golden hamsters expressing hACE2 are susceptible to pangolin sarbecoviruses

Previous studies demonstrated that MpCoV-GX could infect BALB/c, C57BL/6J, and hACE2 transgenic mice but showed limited pathogenicity, with only mild peribronchial and perivascular inflammatory cell infiltration in the lungs (12, 34). Two other studies on MpCoV-GD suggested that the virus exhibited effective infectivity in hACE2 transgenic mice and wild-type hamsters and provided evidence for its potential for transmission (11, 13). In this study, we used SARS-CoV-2, MpCoV-GD, and MpCoV-GX to experimentally infect K18-hACE2 hamsters to assess the variations in pathogenicity among these viruses. Body weight changes, viral load dynamics, and histopathological alterations in lung tissue post-infection were evaluated.

**Fig 4:**
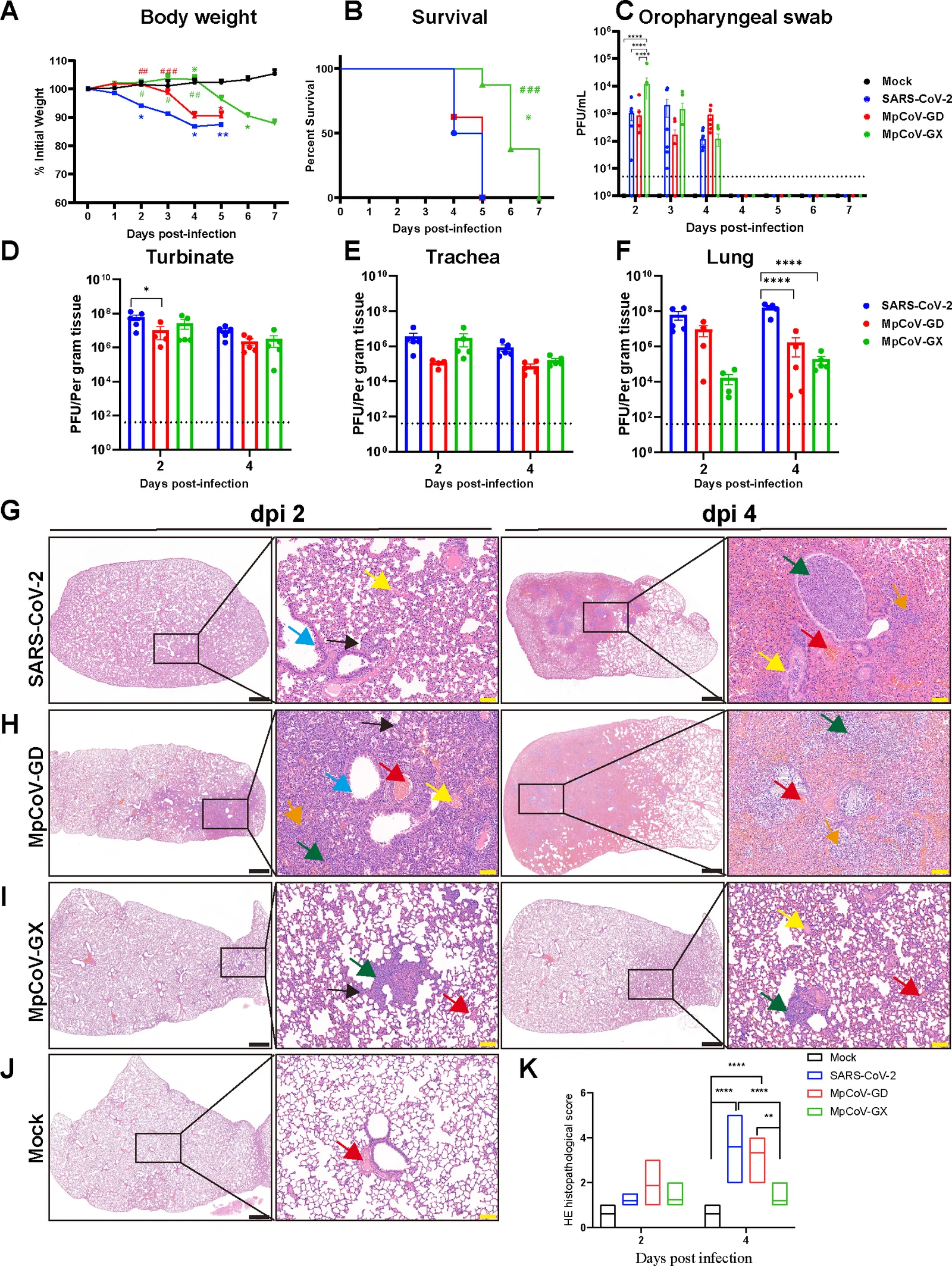
Main characteristics of K18-hACE2 hamster post SARS-CoV-2, MpCoV-GD, and MpCoV-GX infection. Eight- to 12-week-old K18-hACE2 hamsters were mock infected (*n* = 8) or intranasally infected with 1 × 10^5^ TCID_50_ of SARS-CoV-2 (*n* = 20), MpCoV-GD (*n* = 18), or MpCoV-GX (*n* = 18). Body weight (**A**), mortality (**B**), and viral shedding (SARS-CoV-2, *n* = 10; MpCoV-GD, *n* = 8; MpCoV-GX, *n* = 8) (**C**) were monitored every day up to 7 days post-infection (dpi). Viral titers in the turbinate (**D**), trachea (**E**), and lungs (**F**) of infected hamsters were detected by plaque assay (*n* = 5). The dotted line indicates the limit of detection in the plaque assay. Lung pathological changes of K18-hACE2 hamsters infected with SARS-CoV-2 (**G**), MpCoV-GD (**H**), MpCoV-GX (**I**), or DMEM-Mock (**J**) were analyzed. Arrow definitions indicating histopathological changes in hamster lung tissues: blue arrow, localized alveolar wall thickening, or tracheobronchiolar epithelial cell shedding; orange arrow, extensive inflammatory cell infiltration within the alveolar cavity; green arrow, peritracheal granulocyte infiltration; yellow arrow, alveolar architectural disruption and tissue fluid accumulation and minor fibrin exudation; red arrow, enhanced vascular congestion and capillary dilation; black arrow, alveolar wall thickening with monocyte and lymphocyte infiltration. (**K**) Semi-quantitative histopathological evaluation of lung injury in K18-hACE2 hamsters infected with SARS-CoV-2, MpCoV-GD, or MpCoV-GX. Pulmonary lesions were blindly scored on a 0–5 scale evaluating inflammation, alveolar septal thickening, peribronchial/perivascular infiltrates, epithelial loss, consolidation, and bronchial blockage. Grade 0: normal lung with no inflammation or thickening. Grade 1: mild diffuse infiltrates, no alveolar thickening. Grade 2: extensive inflammation, one- to twofold alveolar thickening, moderate peribronchial/perivascular lesions. Grade 3: severe inflammation, focal two- to threefold alveolar thickening. Grade 4: severe injury, epithelial shedding, <10% consolidation, partial bronchial obstruction. Grade 5: severe injury, >10% consolidation, complete bronchial occlusion. Histopathological scores were quantified and compared among groups. Images were acquired by the Pannoramic MIDI system and analyzed by CaseViewer 2.4. Black scale bar, 1,000 μm; yellow scale bar, 100 μm. The errors are presented as mean ± SEM. Body weight was assessed by mixed-effects model. Blue *, SARS-CoV vs Mock; red *, SARS-CoV-2 vs MpCoV-GD; green *, SARS-CoV-2 vs MpCoV-GX; red #, SARS-CoV vs MpCoV-GD; green #, SARS-CoV-2 vs MpCoV-GX; green ⋇, MpCoV-GD vs MpCoV-GX (**A**). Survival curves were analyzed by the Kaplan-Meier method, and statistical significance was determined using the log-rank (Mantel-Cox) test (**B**). Statistical significance was measured by two-way ANOVA (**C–F**). Asterisks denote significant differences (**P < 0.01, ****P < 0.0001); groups without asterisks show no statistical significance.

K18-hACE2 hamsters infected by the three viruses showed progressively declined weight from day 1 for SARS-CoV-2 and days 3 and 5 for MpCoV-GD and MpCoV-GX, respectively (Fig. 4A). The mortality reached 100% for SARS-CoV-2 and MpCoV-GD by day 5 and MpCoV-GX by day 7 (Fig. 4B). To monitor viral shedding, oral swabs were collected from infected hamsters and subjected to plaque assays (Fig. 4C through F). Viral particles were detectable in oral swabs and all tested tissues up to 4 dpi in animals infected by all three viruses (Fig. 4C). MpCoV-GD and MpCoV-GX replication in the turbinate and trachea was comparable to that of SARS-CoV-2 but was significantly weaker than SARS-CoV-2 in lung tissue, particularly at 4 dpi (Fig. 4D through F).

Histopathological changes in lung tissues of infected animals were compared. In SARS-CoV-2-infected lung tissues, localized alveolar wall thickening and shedding of epithelial cells in the trachea and bronchioles (blue arrow) were observed at 2 dpi. Significant infiltration of inflammatory cells was observed within the alveolar cavity (orange arrow), with a notable presence of granulocytes around the tracheal cavity (green arrow). Disruption of alveolar architecture and tissue fluid accumulation were also observed in some alveolar cavities (yellow arrow). Enhanced vascular congestion and capillary dilation were evident (red arrow) (Fig. 4G). Histopathological changes following MpCoV-GD infection were similar to what was observed with SARS-CoV-2 infection (Fig. 4H). MpCoV-GX infection resulted in a few thickened alveolar walls with monocyte and lymphocyte infiltration (black arrow), an increase of macrophages and lymphocytes in some alveolar spaces (green arrow), and minor fibrin exudation (yellow arrow) (Fig. 4I and J). Overall, these results demonstrated comparable lung pathogenicity of MpCoV-GD and SARS-CoV-2 in K18-hACE2 hamsters, which shows more severe pathological changes than the infection by MpCoV-GX (Fig. 4K).

**Fig 5:**
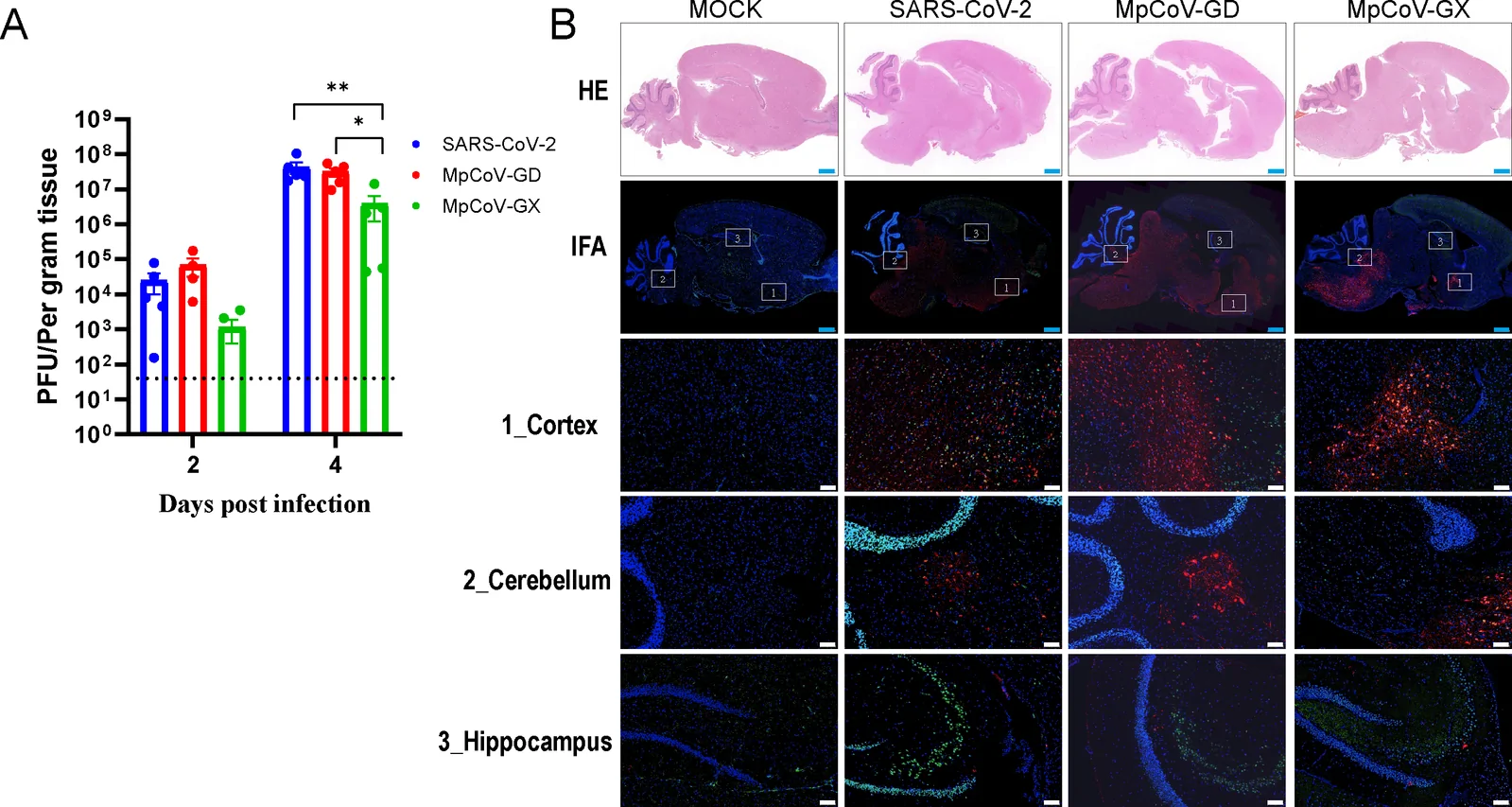
K18-hACE2 hamster brain tissue was infected with SARS-CoV-2, MpCoV-GD, and MpCoV-GX. (**A**) Hamsters were humanely euthanized at 4 dpi. Virus titers in brain tissues of infected hamsters were quantified via plaque assay (*n* = 5). The dotted line denotes the lower limit of detection of the plaque assay. (**B**) Brain tissue sections were stained to demarcate discrete brain regions. Viral NP antigen was stained red, and neurons were stained green. Row 1, panoramic HE-stained brain sections; row 2, IFA staining; row 3, magnified cerebral cortex; row 4, cerebellum; row 5, hippocampus. Images were collected using FV1200 confocal microscopy. The blue scale bar is 1,000 μm, and the white scale bar is 100 μm. The errors are presented as mean ± SEM. Statistical significance was measured by two-way ANOVA (**A**). Asterisks denote significant differences (*P < 0.05, **P < 0.01); groups without asterisks show no statistical significance.

In addition to respiratory symptoms, neurological manifestations including impaired consciousness and headache have been reported in some COVID-19 patients as well (35). Animal infection experiments have demonstrated that the host ACE2 is required for SARS-CoV-2 infection of neurons (36). Thus, overexpression of hACE2 in the transgenic mouse resulted in the neuroinvasion of SARS-CoV-2. The onset of severe disease in infected hamsters was correlated with the peak levels of viruses in the brain and death of central nervous system (CNS) cells (Fig. 5A). SARS-CoV-2 and MpCoV-GD/-GX virus antigen was detected throughout the brain including the cortex and hippocampus, indicating efficient replication of the pangolin sarbecoviruses in the brains of K18-hACE2 hamsters (Fig. 5B). These results are consistent with previous studies of SARS-CoV-2 in K18-hACE2 mice (37, 38).

### Transmission of pangolin sarbecoviruses in K18-hACE2 hamsters

**Fig 6:**
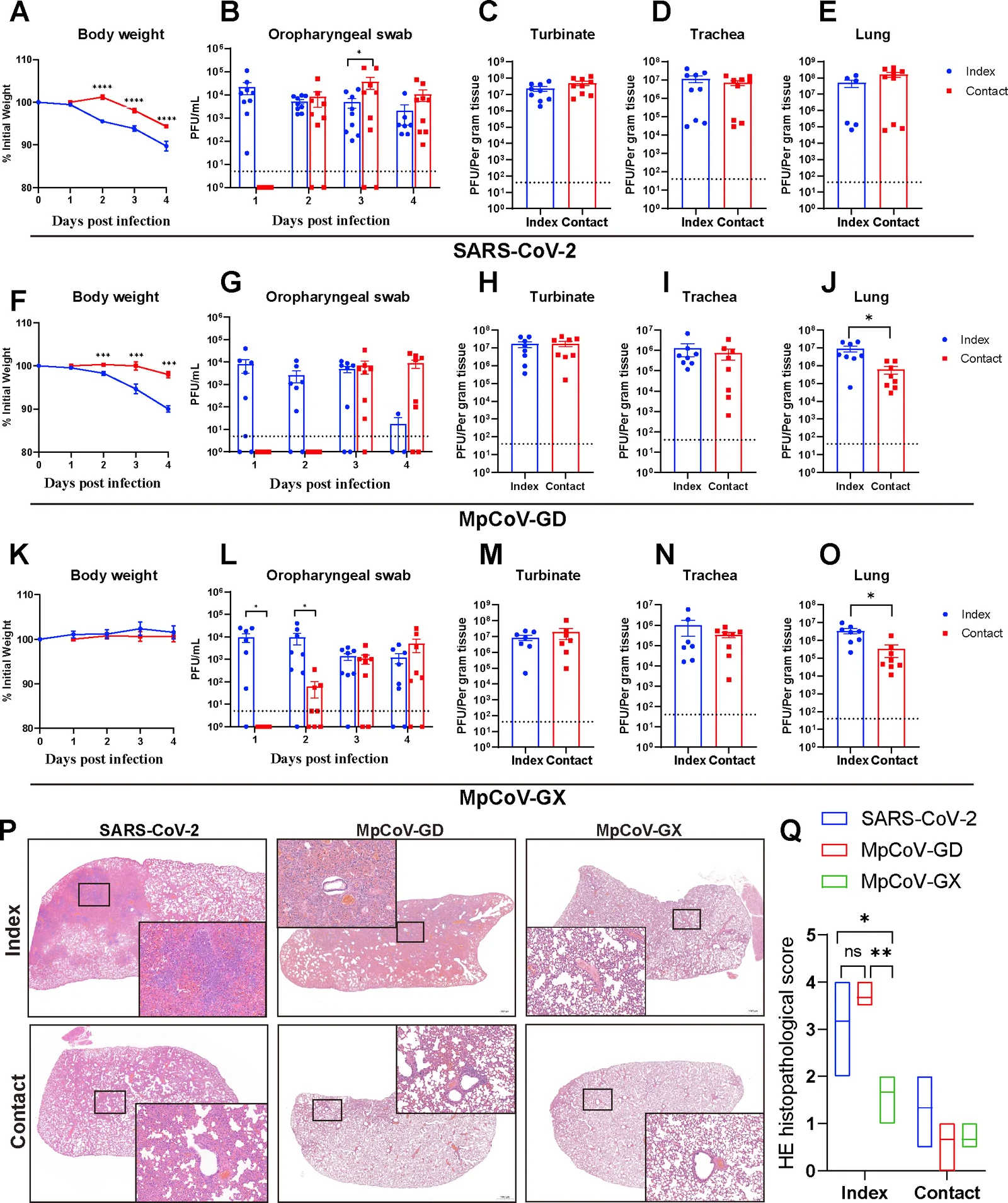
Directed contact transmission of SARS-CoV-2, MpCoV-GD, and MpCoV-GX in K18-hACE2 hamsters. Eight- to 12-week-old K18-hACE2 hamsters were intranasally infected with 1 × 10^5^ TCID_50_ of SARS-CoV-2 (*n* = 9), MpCoV-GD (*n* = 8), or MpCoV-GX (*n* = 8). Twenty-four hours later, each inoculated animal (index) was co-housed with one naive hamster (contact). Body weight changes were monitored (**A**, **F,** and **K**) and statistically analyzed using a mixed-effects model. Viral shedding in oropharyngeal swabs was monitored up to 4 dpi (**B, G**, and **L**). Viral titers in the turbinate (**C**, **H**, and **M**), trachea (**D**, **I, **and **N**), and lungs (**E, J**, and **O**) of infected or contact-infected hamsters at 4 dpi were determined by plaque assay, and pathological changes in lungs were analyzed by H & E staining (**P**). The dotted line indicates the limitation of detection in plaque assay. (**Q**) Semi-quantitative scoring of lung histopathological lesions in index and contact hamsters. The errors are presented as mean ± SEM. Statistical significance was measured by unpaired Student’s *t*-test (**J** and **O**) and two-way ANOVA (**B**, **G**, **L, **and **Q**). Asterisks denote significant differences (*P < 0.05, **P < 0.01); groups without asterisks show no statistical significance. Images were acquired by the Pannoramic MIDI system and analyzed by CaseViewer 2.4. Scale bar, 1,000 μm.

Human coronaviruses mainly transmit through respiratory droplets. However, aerosol, direct contact with contaminated surfaces, and fecal-oral transmission were also reported during the SARS epidemic and COVID-19 (39–41). Previous studies have shown that MpCoV-GX and MpCoV-GD can be horizontally transmitted among wild-type hamsters through direct contact (11, 13, 14). Nevertheless, the relative transmissibility of these different viruses has not been investigated. Here, we compare the transmission of SARS-CoV-2, MpCoV-GD, and MpCoV-GX in K18-hACE2 hamsters. Hamsters were intranasally infected with 1 × 10^5^ TCID_50_ of SARS-CoV-2, MpCoV-GX, or MpCoV-GD and kept in individual cages. After 24 h, each inoculated animal (index) was co-housed with one naive hamster. Body weight was recorded, and oral swabs were collected daily. Hamsters infected with SARS-CoV-2 showed weight loss upon infection, while contacted hamsters showed weight loss from day 2 dpi (Fig. 6A). High titers of SARS-CoV-2 were continuously detected in throat swabs of all index animals throughout the testing period. Among the contacted animals, viable virus could be found in throat swabs from only one hamster on days 2 and 3 post-infection, while all hamsters had detectable levels of SARS-CoV-2 live virus by day 4 post-infection (Fig. 6B). Moreover, robust viral titers were detected in nasal turbinates, tracheae, and lungs of all index and contacted hamsters at 4 dpi (Fig. 6C through E). Hamsters infected with MpCoV-GD exhibited a relatively slow rate of weight loss (Fig. 6F). In the index hamsters, MpCoV-GD could be detected in throat swabs every day, whereas it can only be detected in one of the four contacted hamsters on days 3 and 4 (Fig. 6G). Live virus of MpCoV-GD could be detected in the turbinate, trachea, and lung tissues of both index and contacted hamsters, while virus titer in contacted hamster lung was significantly lower than that of index hamsters (Fig. 6H through J). Neither index nor contacted hamsters showed weight loss within 4 days after infection with MpCoV-GX (Fig. 6K), but the virus shedding is similar to that of SARS-CoV-2 (Fig. 6L). Proliferation of MpCoV-GX can also be observed in the turbinate, trachea, and lung tissues of index and contacted hamsters, whereas contacted hamster lung harbors a fewer infectious viruses (Fig. 6M through O). For all the three viruses, hamsters infected through contact developed mild lung damage, showing only localized alveolar wall thickening (Fig. 6P). There was no significant pathological scoring difference among the three viruses in contacted hamsters (Fig. 6Q).

**Fig 7:**
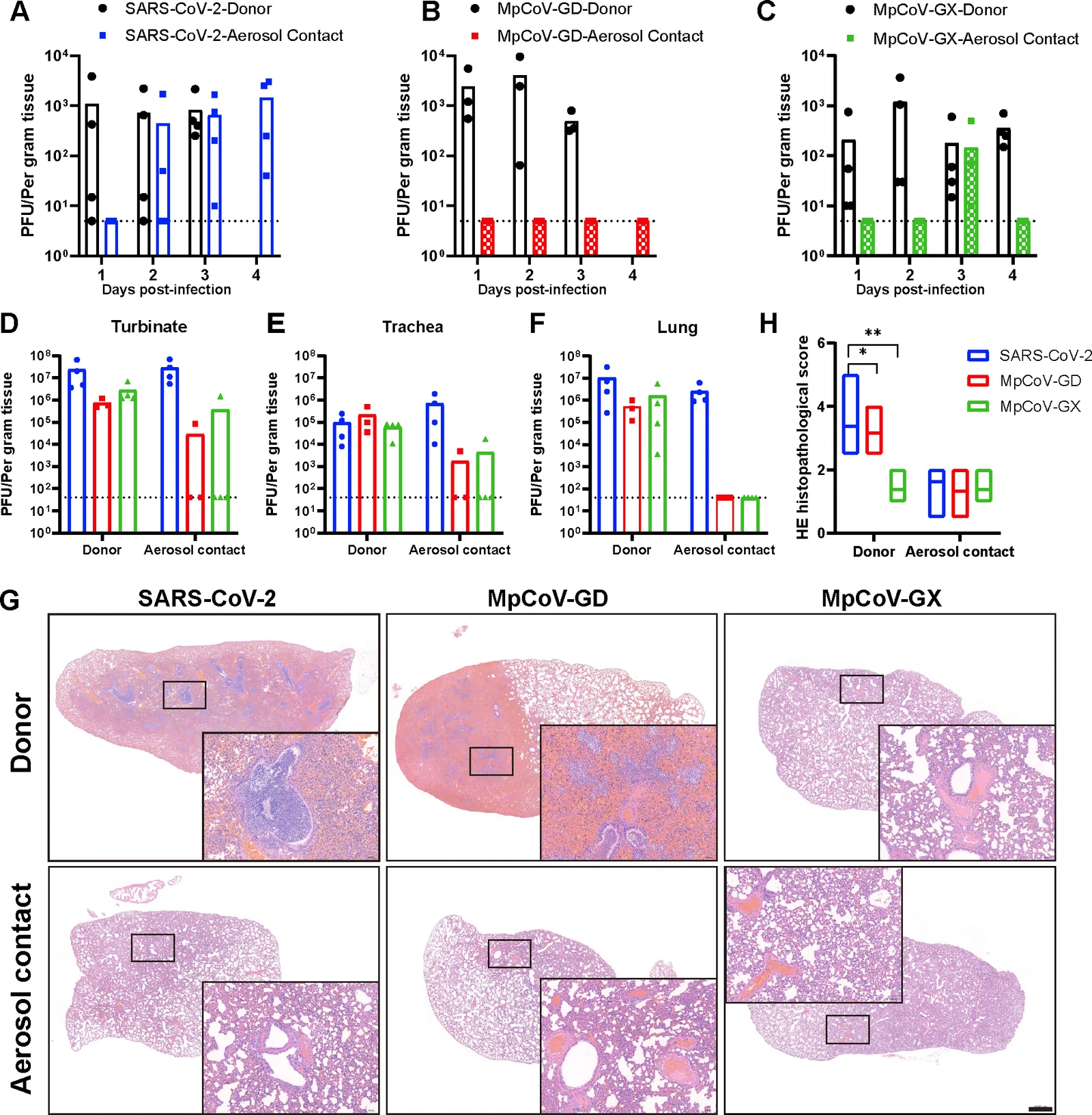
Transmission of SARS-CoV-2 in K18-hACE2 hamsters via aerosol. (**A–C**) Infectious viral titer in the oral swabs of donor hamsters inoculated with 1 × 10^5^ TCID_50_ of SARS-CoV-2, MpCoV-GD, or MpCoV-GX and aerosol contact hamsters (SARS-CoV-2 and MpCoV-GX, *n* = 4; MpCoV-GD, n = 3) was determined by the plaque assay. (**D–F**) Viral titers in the turbinate, trachea, and lungs of the donor and aerosol contact hamsters at 4 dpi, determined by plaque assay. The dotted line indicates the limit of detection in plaque assay. (**G**) Pathological changes in the lungs of K18-hACE2 hamsters following SARS-CoV-2, MpCoV-GD, and MpCoV-GX infection or aerosol infection at 4 dpi. (**H**) Semi-quantitative scoring of lung histopathological lesions in donor and aerosol contact hamsters. Statistical significance was measured by two-way ANOVA. Asterisks denote significant differences (*P < 0.05, **P < 0.01); groups without asterisks show no statistical significance.

We further compared the efficiency of aerosol transmission of the three viruses among K18-hACE2 hamsters. Donor hamsters were intranasally infected with 1 × 10^5^ TCID_50_ of SARS-CoV-2, MpCoV-GD, or MpCoV-GX and then housed in a mesh cage. Each infected hamster was housed adjacent to an uninfected recipient hamster in another mesh cage to acquire airborne transmission. Cages were placed in a glove compartment which served as an isolator. The spacing between the cages is 15 cm, and the airflow within the isolator is directed from the infected group toward the uninfected group to facilitate aerosol dispersion. Viral shedding from respiratory secretions in hamsters exposed to SARS-CoV-2 infection aerosols was detected from day 2 to day 4 post-exposure, suggesting efficient aerosol transmission of SARS-CoV-2 (Fig. 7A). For the groups of pangolin sarbecoviruses, virus excretion was detected in three of four contacted hamsters following exposure to MpCoV-GX at day 3 (Fig. 7B and C). Although high viral titers were detected in the turbinate and trachea, the virus loads were undetectable in lung tissues of all animals exposed to MpCoV-GD- and MpCoV-GX-infected animals (Fig. 7D through F). These results indicate the lower aerosol transmission efficiency of these two pangolin viruses compared to SARS-CoV-2. Furthermore, the histopathological alterations in lung tissue were similar to those observed in experiments of contact transmission (Fig. 7G and H). Together, these data suggest that the pangolin sarbecoviruses may transmit via aerosol but with low efficiency.

### Potential therapeutics against infection of pangolin sarbecoviruses

**Fig 8:**
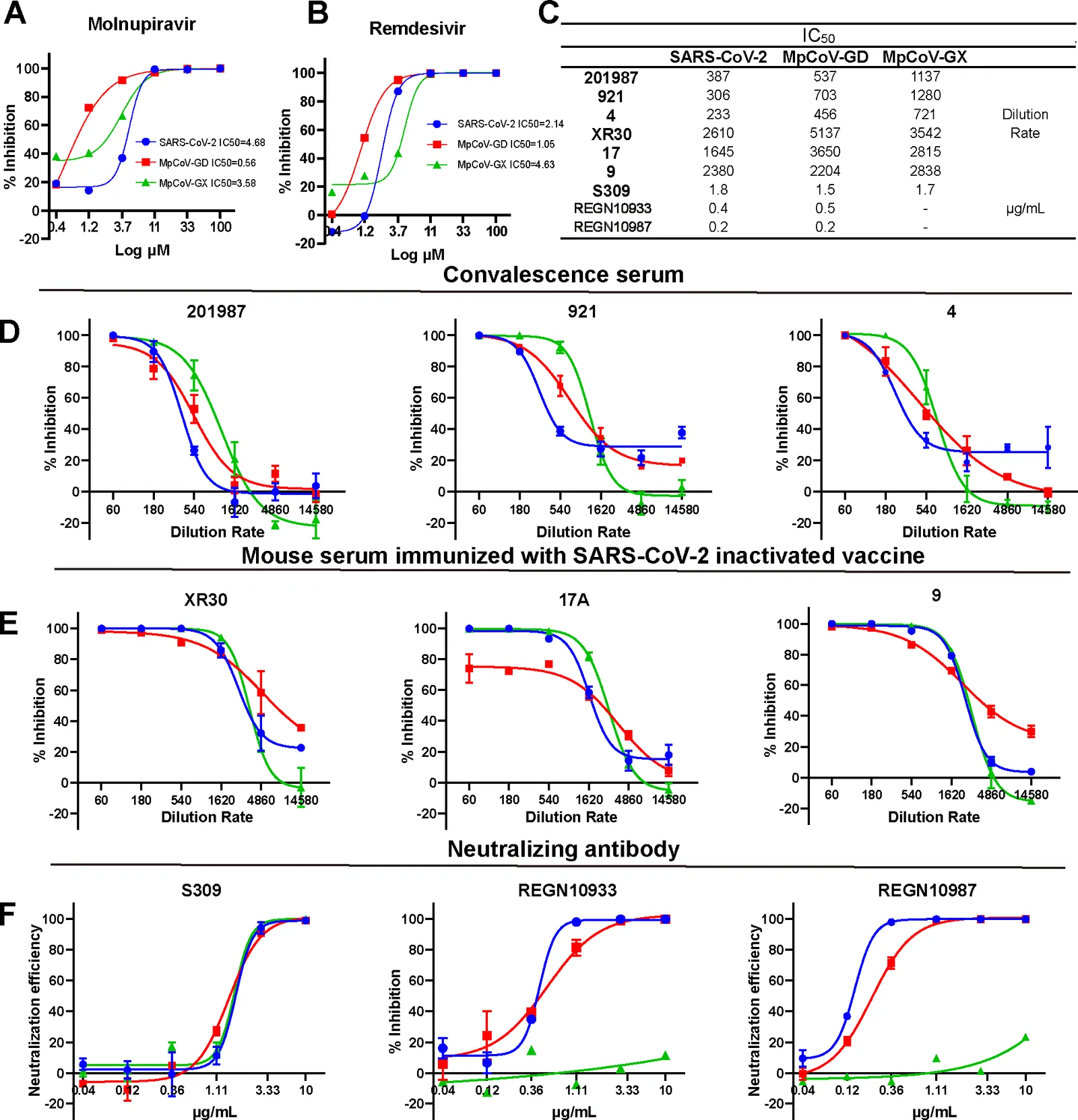
Inhibition of pangolin SARS-CoV-2-related viruses by monoclonal antibodies, antiviral drugs, and serum from convalescent patients against SARS-CoV-2 VeroE6 cells was infected with SARS-CoV-2, MpCoV-GD, or MpCoV-GX at an MOI of 0.01 and treated with different doses of molnupiravir (**A**) and remdesivir (**B**) for 24 h, and viral yield in the cell supernatant was subsequently collected and quantified using qRT-PCR. The neutralizing effect of convalescent patient serum (**D**), mouse serum immunized with a SARS-CoV-2 inactivated vaccine (**E**), or SARS-CoV-2 monoclonal antibodies (**F**) against SARS-CoV-2, MpCoV-GD, and MpCoV-GX was quantified using a plaque assay. (**C**) A summary table presenting IC_50_ values for the evaluation of antibodies, drugs, and sera against SARS-CoV-2, MpCoV-GD, or MpCoV-GX.

The above results reveal that pangolin sarbecoviruses can infect human cells and organoids and exhibit pathogenicity in the humanized ACE2 hamster model. Hence, there is a need to prepare effective therapeutics and prophylactic agents such as antiviral drugs and neutralizing antibodies against these viruses. Here, we evaluated the inhibitory effects of antiviral medications against SARS-CoV-2 on MpCoV-GD and MpCoV-GX. The results showed that two FDA-approved SARS-CoV-2 drugs, molnupiravir and remdesivir, had inhibitory effects on both MpCoV-GD and MpCoV-GX (Fig. 8A and B). Next, we used convalescent sera from COVID-19 patients with high antibody titers and sera from mice immunized with inactivated SARS-CoV-2 vaccine to test their neutralizing effects on MpCoV-GD and MpCoV-GX. The results indicated that they all had potent neutralizing effects on the two pangolin coronaviruses (Fig. 8D and E), suggesting that immunization with inactivated SARS-CoV-2 vaccine can offer a certain degree of protection against wildlife-borne sarbecoviruses. We also evaluated whether SARS-CoV-2 monoclonal antibodies elicited cross-protection against pangolin sarbecoviruses. The monoclonal antibody S309 demonstrated broad-spectrum neutralizing capabilities and could effectively neutralize MpCoV-GD and MpCoV-GX. However, REN10933 and REN10987 only showed neutralizing effects on MpCoV-GD and hardly neutralized MpCoV-GX (Fig. 8F). We have summarized the inhibitory effects of tested sera and antibodies on MpCoV-GD and MpCoV-GX in Fig. 8C.

## DISCUSSION

Pangolins were not considered important reservoirs or intermediate hosts for viruses until the detection of SARS-CoV-2-related CoVs in these animals smuggled to China from Southeast Asia (8, 9). In addition to coronaviruses, highly diverse viruses have been found in deceased pangolins through next-generation sequencing (42). These studies suggest that pangolins are highly susceptible hosts to viruses and pose a potential risk of zoonotic infectious diseases. However, how the viruses were transmitted to and evolved in pangolins and the role of pangolins played in the transmission of viruses to humans are largely unknown. In the present study, we performed a biological characterization of two pangolin SARS-CoV-2-related viruses in comparison with the SARS-CoV-2 prototype strain *in vitro* and *in vivo*. Compared with SARS-CoV-2, the two pangolin CoVs show attenuated infection and replication in human cell lines and organoids, as well as lower pathogenicity and transmission capacity in K18-hACE2 hamsters.

Two pangolin SARS-CoV-2-related viruses are highly divergent among themselves and with SARS-CoV-2 in full-length genome sequences and spike proteins. However, both of them have a similar efficiency in binding and utilizing human ACE2 for entry as SARS-CoV-2 (32). In this study, we have a detailed comparison in entry and replication efficiency of the three sarbecoviruses in cell lines from monkeys and humans as well as in human organoids. In the tested cells, host proteases can enhance the entry of SARS-CoV-2 prototype strain and the two pangolin viruses which is in line with previous studies (32). The three viruses all employ the membrane fusion and endocytosis entry routes for entering the cells. SARS-CoV-2 has a little bit higher membrane fusion efficiency than the MpCoV-GD, followed by MpCoV-GX. Consistently, SARS-CoV-2 and MpCoV-GD have an overall higher entry efficiency than MpCoV-GX in terms of attachment and internalization capacity. However, the one-step growth curves showed that SARS-CoV-2 and MpCoV-GX replicate better than MpCoV-GD in the four cell lines and human organoids except in nasal organoids. These results indicate that although MpCoV-GD has a higher entry efficiency, it cannot overcome host restriction to accomplish the life cycle. On the contrary, MpCoV-GX has a lower entry efficiency, but likely has a better adaptation in replication in human cells. Overall, these two pangolin viruses have not adapted to humans by showing either relatively low replication efficiency or low entry efficiency in human cell lines and organoids. To eliminate inter-individual variations and further investigate the infectivity and pathogenic risks of the virus in humans, future in-depth research utilizing organoids derived from a broader diversity of human donors is warranted.

In previous *in vivo* studies, the pathogenicity of MpCoV-GX and MpCoV-GD was separately examined via infection in hACE2 transgenic or knock-in mice or wild-type golden hamsters, and their potential of contact transmission or airborne transmission was evaluated using wild-type hamsters (11–14). Here, we assessed the cross-species infection risk of the two pangolin viruses by investigating pathogenicity and transmissibility in a newly developed humanized animal model, the K18-hACE2 hamster model. All the three viruses caused body weight loss and eventually 100% mortality in K18-hACE2 hamsters, with the MpCoV-GX showing a delayed mortality. High viral load was detected in the turbinate, trachea, and lungs of the infected animals. SARS-CoV-2 and MpCoV-GD induced similar pathological changes in hamster lung tissues, which were more severe than those observed in the MpCoV-GX-infected group. In addition to the respiratory tract, high replication level of the three viruses was also detected in hamster brains. Therefore, the fatal outcome of the virus infection in these hamsters is likely a result of brain infection and may not be a real-world reflection of their infection features. In contrast to cell culture phenotypes, *in vivo* pathogenicity and tissue tropism of the two pangolin sarbecoviruses were more closely associated with their spike protein homology to SARS-CoV-2. MpCoV-GD shared high RBD sequence similarity with SARS-CoV-2, enabling faster brain invasion and more severe clinical manifestations compared with MpCoV-GX. The contrasting patterns between *in vitro* replication fitness and *in vivo* pathogenicity suggest that the spike protein sequence may primarily determine viral tropism and pathogenic potential *in vivo*, whereas viral replication efficiency in cultured cellular system is likely shaped by the non-spike genomic backbone elements. Using K18-hACE2 hamsters, we further compared the transmission ability of the three viruses via direct contact and aerosol transmission. We demonstrated the two animal viruses are able to transmit via direct contact and limited aerosol transmission but with a much lower transmissibility compared with SARS-CoV-2. Immunization with a pre-existing vaccine or administration of broad-spectrum antivirals that elicit cross-protection is an effective strategy for promptly impeding the spread of emerging infectious diseases (23, 43, 44). Previous studies revealed that two RdRp-targeting antivirals, Remdesivir and Molnupiravir, can broadly inhibit propagation of a wide range of coronaviruses *in vitro* (45, 46). In this study, we found that the two drugs effectively inhibit the replication of MpCoV-GD and MpCoV-GX as SARS-CoV-2 (Fig. 8). The sequence conservation of RdRp among different coronaviruses may account for the broad antiviral activity of these molecules. Two COVID-19 therapeutic monoclonal antibodies, namely, REGN10933 and REGN10987, were previously shown to effectively prevent and treat SARS-CoV-2 infection in rhesus macaques and hamsters (47, 48). In our study, these two monoclonal antibodies displayed efficient neutralizing activity against MpCoV-GD but not MpCoV-GX. The result aligns well with a previous study in which a monoclonal antibody commercially known as etesevimab efficiently neutralized MpCoV-GD but not MpCoV-GX (49). As these antibodies target the RBD, the difference in the neutralization effect on the two viruses is likely due to higher sequence identity and antigenic similarity of MpCoV-GD to SARS-CoV-2. Sequence alignment of RBD epitopes provides a molecular basis for interpreting the failure of neutralization of MpCoV-GX by REGN10933 and REGN10987. MpCoV-GX displays marked amino acid alterations at both the REGN10987 lateral loop epitope and the REGN10933 RBM epitope (50), whereas these two binding regions are well-conserved between SARS-CoV-2 and MpCoV-GD. For MpCoV-GX, extensive substitutions at the core antibody-interacting residues impair antibody-RBD binding, which may contribute to the neutralization escape. Our and others’ studies have shown that convalescent sera from COVID-19 patients and sera from mice immunized with SARS-CoV-2 vaccines can effectively neutralize both MpCoV-GD and MpCoV-GX though the neutralization potency is reduced as compared to that against SARS-CoV-2 (11, 13, 49). These results indicate that high titers of serum antibody induced by whole-virus of SARS-CoV-2 may provide protection against infection of the pangolin sarbecoviruses. It implies that whole-virus immune sera can be a viable approach for broad-spectrum treatment of emerging sarbecoviruses. Together, our data provide guidance for the design and development of pan-sarbecovirus preventive or therapeutic candidates for combating future SARS-like diseases.

Bats are recognized as natural reservoirs of a great diversity of sarbecoviruses, while pangolins have also drawn our attention as two distinct sarbecoviruses have been recently identified from these animals. However, these groups of coronaviruses are not widely occurring among pangolins, suggesting that they are more likely to be intermediate hosts. Besides sarbecoviruses, we have identified a pangolin MERS-like coronavirus which is likely to have originated from a bat HKU4 coronavirus, but with enhanced human DPP4 receptor binding affinity, further supporting the plausibility that pangolins may act as intermediate hosts of bat coronaviruses and play important roles in their interspecies transmission and adaptation (51). Furthermore, metagenomic analyses of smuggled pangolins have unveiled the presence of a great diversity of novel viruses belonging to other families. Sequences of some viruses showed similarity to human-associated viruses, such as respiratory syncytial virus and rotavirus A, suggesting that pangolins may carry numerous other viruses with zoonotic transmission risk (42, 52). Thus, there is a strong need to conduct further virus surveillance among these animals to better understand the evolution and transmission of emerging viruses from wildlife.

In conclusion, we have performed a comprehensive comparison of the cell entry, replication, pathogenicity, and transmissibility of MpCoV-GD and MpCoV-GX. Our findings demonstrate the potential cross-species infection risk of these two pangolin sarbecoviruses though they have attenuated infectivity, pathogenicity, and transmissibility as compared to SARS-CoV-2. Efforts should be made to strengthen pathogen surveillance among pangolins and other potential intermediate host of zoonotic viruses, as well as to prepare countermeasure against future disease emergence.

## Data Availability

The genome sequence of MpCoV-GX is publicly available in GenBank under accession number MT072864.1. The genome sequence of MpCoV-GD is available in GenBank under accession number MT121216.1. MpCoV-GD was recovered from a full-length cDNA clone constructed using yeast-based genetic engineering technology in this study.

## AFTER EPUB

*[This article was published on 24 September 2026 with an error in the title. The title was corrected in the current version, posted on 1 October 2026.]*
